# Cytokine profiling of umbilical cord blood plasma

**DOI:** 10.1186/1710-1492-10-S1-A61

**Published:** 2014-03-03

**Authors:** Katrina K Au, Jenny Thiele, Anne K Ellis

**Affiliations:** 1 Department of Biomedical Sciences, Queen’s University, Kingston, Ontario, K7L 3N6, Canada; 2 Department of Medicine, Queen’s University, Kingston, Ontario, K7L 3N6, Canada

## Background

Cytokines have been shown to be important signal molecules in the development of allergy and asthma, especially the regulatory cytokine IL-10, which has been shown to correlate with higher risk of allergy development in children [1]. Research results regarding cytokine levels in umbilical cord blood plasma vary greatly; some studies find the concentration of cytokines detectable whereas other studies do not. The purpose of this pilot study was to determine if cytokines could be measured from cord blood plasma using IL-10 ELISAs and xMAP Luminex assay.

## Methods

Umbilical cord blood was collected into EDTA vacutainer tubes (BD) from mothers who underwent an elective Caesarean section at Kingston General Hospital and gave written informed consent. Twenty minutes post collection, plasma was separated by centrifugation at 1300 g for 10 minutes and 500 μl aliquots were temporarily stored at -80 °C. One aliquot was assayed using the human IL-10 ELISA (EBiosciences). In a follow up analysis, a second plasma sample aliquot was examined using the Milliplex map kit (Millipore) that targeted human IL-1b, IL-2, IL-4, IL-5, IL-6, IL-7, IL-8, IL-10, IL-12(p70), IL-13, IFN-γ, GM-CSF, and TNF-α.

## Results

The IL-10 ELISA showed that IL-10 was present in 1 of 6 the plasma samples examined. Using the Milliplex assay, IL-1b was detectable in 60% of the samples; IL-2 in 50% of the samples; IL-4 and IL-5 in 90% of the samples; IL-6, IL-7 and IL-8 in 100% of the samples; IL-10 in 90% of the samples; IL-12(p70) in 80% of the samples; IL-13 in 100% of the samples; IFN-γ and GM-CSF in 80% of the samples; TNF-α in 100% of the samples.

## Conclusions

The ELISA’s lower detection limit of 2 pg/ml was not sensitive enough to measure IL-10 accurately in these cord blood plasma samples. The lower limit of detection of the Milliplex map kit assay ranged from 0.01 pg/ml for IL-5 to 0.48 pg/ml for IL-13, and was sufficient to determine the cytokines concentrations in most of the samples. The variation observed in the measurement of the cytokine levels may have been due to the hemolysis of two of the samples as well as the samples being stored at -80 °C instead of -20 °C. Future research will compare cytokine levels in umbilical cord blood plasma from atopic mothers compared to non-atopic mothers.
